# Effects of age on symptom burden, mental health and quality of life amongst people with HIV in the UK

**DOI:** 10.7448/IAS.17.4.19511

**Published:** 2014-11-02

**Authors:** Jennifer McGowan, Lorraine Sherr, Alison Rodger, Martin Fisher, Alec Miners, Margaret Johnson, Jonathan Elford, Simon Collins, Graham Hart, Andrew Phillips, Andrew Speakman, Fiona Lampe

**Affiliations:** 1 Infection and Population Health, University College London, London, UK; 2 HIV Medicine, Brighton and Sussex University Hospitals NHS Trust, Brighton, UK; 3 Department of Health Services Research and Policy, London School of Hygiene and Tropical Medicine, London, UK; 4 HIV Medicine, The Royal Free Centre for HIV Medicine, London, UK; 5 Evidence-based Health Care, City University, London, UK; 6 HIV i-Base, London, UK; 7 Population Health Sciences, University College London, London, UK

## Abstract

**Introduction:**

The evolving HIV epidemic, coupled with advances in HIV treatment, has resulted in an ageing HIV-diagnosed population. It has been suggested that adverse physical and psychological effects of HIV may be higher among older people. However, few studies have examined the effect of older age on well-being for people with HIV.

**Materials and Methods:**

The ASTRA study included 3258 HIV-diagnosed individuals (2248 MSM; 373 heterosexual men; 637 women) recruited from eight UK clinics in 2011–12 (64% response rate). Participants completed a questionnaire that included standard inventories on symptoms and health-related quality of life (HrQoL). Associations of age group with: physical symptom distress (reporting significant distress for ≥1 of 26 symptoms), depression and anxiety (score ≥10 on PHQ-9 and GAD-7, respectively) and HrQoL problem (reporting problems on ≥1 of 5 Eurqol-5D domains) were assessed; adjustment was made for gender/sexuality and time with diagnosed HIV.

**Results:**

Of all participants, 87% were taking ART, 76% had VL ≤50c/mL and 19% had CD4 <350/mm^3^. Mean age was 45 years (range 18–88) with 5% <30, 23% 30–39, 43% 40–49, 22% 50–59 and 7% ≥60 years. The most prevalent distressing physical symptoms were: lack of energy/tiredness (26%), difficulty sleeping (24%), muscle-ache/joint pain (21%) and pain (18%). With older age, there was no clear trend in prevalence of physical symptom distress, but prevalence of depression and anxiety decreased, while prevalence of HrQoL problems increased. This pattern remained after adjustment for gender/sexuality and time diagnosed with HIV. The increase with age in overall prevalence of HrQoL problem was due to increased problems for “mobility,” “self-care” and “performing usual activities” domains, not an increase in “depression/anxiety.” Longer time with diagnosed HIV was strongly associated with higher prevalence of all symptoms measures and HrQoL problem (*p*<0.001 for trend, adjusted models).

**Conclusions:**

Physical and psychological symptoms are common among people living with HIV, but the burden of these symptoms is not highest among the older age group. While HrQoL tended to worsen with older age, physical symptom distress did not, and mental health improved. This may reflect greater resilience in older adults, or the potential for “successful ageing”: maintaining mental health despite age-related health losses.

**Table 1:** Adjusted association of age with symptom prevalence among PWH

N=3258	Physical symptom distress		Depression		Anxiety		Health-related QoL problem	
Age (years)	%	Adjusted OR* (95% CI)	%	Adjusted OR* (95% CI)	%	Adjusted OR* (95% CI)	%	Adjusted OR* (95% CI)
<30	60	1.6 (1.1, 2.5)	35	3.1 (1.9, 5.1)	28	3.5 (2.0, 6.1)	63	0.9 (0.6, 1.5)
30–39	51	1.1 (0.8, 1.5)	25	1.8 (1.2, 2.7)	19	2.0 (1.2, 3.2)	57	0.7 (0.5, 1.0)
40–49	56	1.1 (0.8, 1.5)	31	2.1 (1.5, 3.1)	23	2.3 (1.5, 3.6)	65	0.8 (0.6, 1.2)
50–59	61	1.3 (1.0, 1.9)	31	2.1 (1.4, 3.1)	23	2.3 (1.4, 3.6)	72	1.1 (0.8, 1.5)
≥60	53	1 (reference)	18	1 (reference)	12	1 (reference)	70	1 (reference)
		p=0.92 for trend		p=0.010 for trend		p=0.012 for trend		p=0.004 for trend

* Odds ratio adjusted for gender/sexuality (MSM, heterosexual men and women) and time with diagnosed HIV (0–2, 2–10 and >10 years) using logistic regression

